# Supplementary material for: Development of an Antigen Capture Lateral Flow Immunoassay for the Detection of Burkholderia pseudomallei
Source: Diagnostics (Basel). 2024 May 16;14(10):1033. doi: 10.3390/diagnostics14101033 (PMC11120185; doi:10.3390/diagnostics14101033)
Supplement: Supplementary file 1 [file diagnostics-14-01033-s001.zip › diagnostics-2955626-supplementary.pdf]

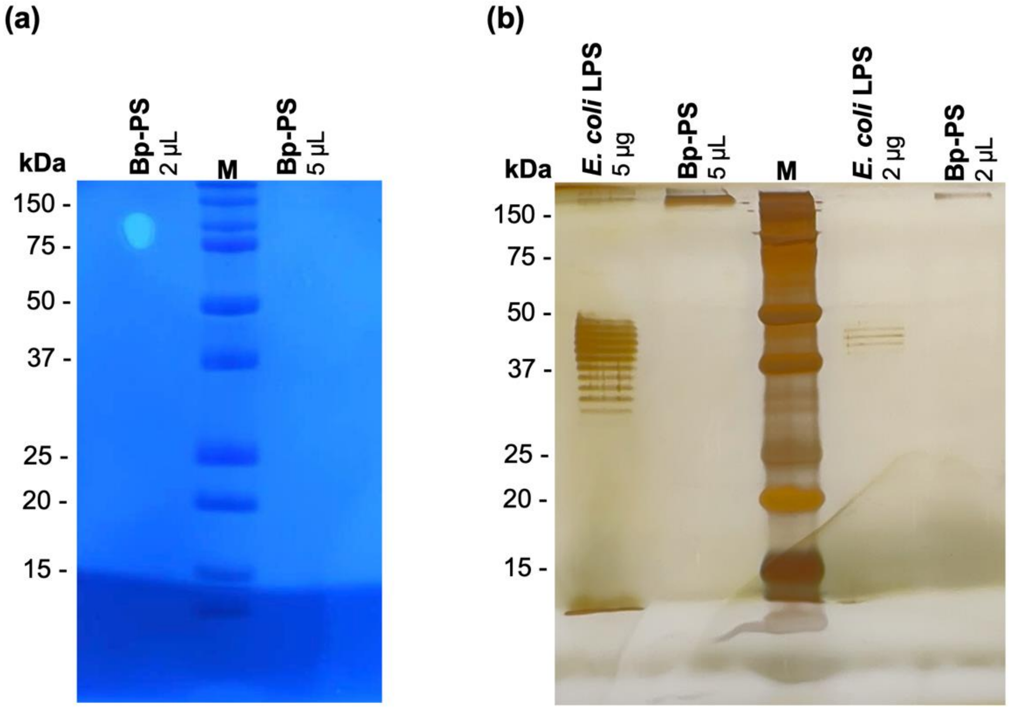

Figure S1. Bp-PS analyzed by SDS-PAGE with Coomassie blue staining (a) and modified silver staining (b). Bp-PS samples were loaded on 15% SDS-PAGE gels at 2 µL and 5 µL per lane. *E. coli* LPS samples were also loaded at 2 µg and 5 µg per lane as controls for the modified silver staining. The SDS-PAGE gel with Coomassie blue staining (a) shows no protein band in Bp-PS samples, indicating that proteins were completely removed. Bp-PS in the SDS-PAGE gel with modified silver staining (b) shows a band (possibly polysaccharide) at the high molecular weight, which corresponds to the size of the CPS. The result suggests that Bp-PS contains mainly CPS.

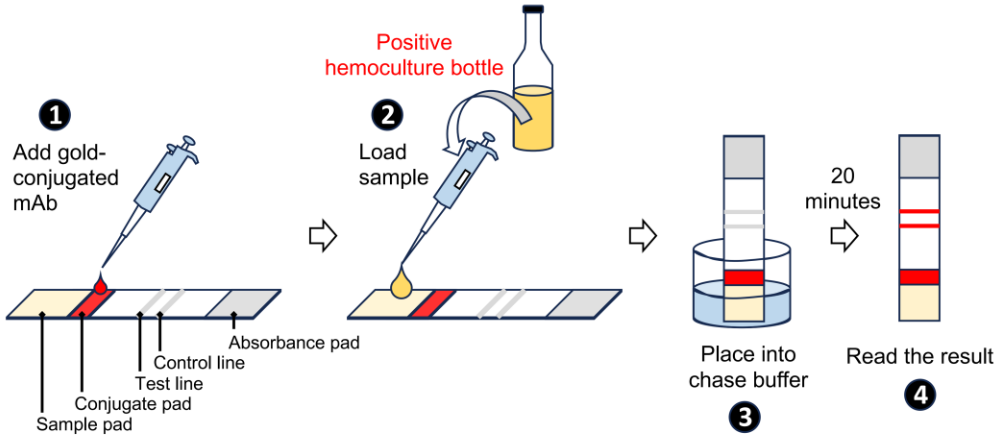

Figure S2. Step-by-step use of the prototype LFIA. The use of the prototype LFIA to detect *B. pseudomallei* consists of four steps: (i) add 4 µL of the gold-conjugated Bp2.1 to the conjugate pad; (ii) load 20 µL of hemoculture medium directly onto the sample pad; (iii) place the LFIA into 200 µL of chase buffer and allow it to flow for 20 minutes; and (iv) inspect the test and control lines visually.
